# Burden of ischemic heart disease mortality attributable to physical inactivity in Brazil

**DOI:** 10.11606/S1518-8787.2018052000413

**Published:** 2018-07-13

**Authors:** Diego Augusto Santos Silva, Deborah Carvalho Malta, Maria de Fatima Marinho de Souza, Mohsen Naghavi

**Affiliations:** I Universidade Federal de Santa Catarina. Núcleo de Pesquisa em Cineantropometria e Desempenho Humano. Florianópolis, SC, Brasil; II Universidade Federal de Minas Gerais. Escola de Enfermagem. Departamento de Enfermagem Materno-Infantil e Saúde Pública. Belo Horizonte, MG, Brasil; III Ministério da Saúde. Departamento de Vigilância de Doenças e Agravos Não Transmissíveis e Promoção da Saúde. Brasília, DF, Brasil; IV University of Washington. Institute for Health Metrics and Evaluation. Seattle, WA, United States

**Keywords:** Sedentary Lifestyle, Global Burden of Disease, Cardiovascular Diseases, mortality, Motor Activity

## Abstract

**OBJECTIVE:**

To analyze if the burden of ischemic heart disease mortality trend attributed to physical inactivity in Brazil differs from the global estimates.

**METHODS:**

Databases from the Global Burden of Disease Study for Brazil, Brazilian states, and global information were used. We estimated the summary exposure value for physical inactivity, the total number of deaths, and the age-standardized death rates for ischemic heart disease attributed to physical inactivity in the years 1990 and 2015, and the population-attributable fraction. Data were presented according to sex.

**RESULTS:**

The Brazilian population was found to have a risk of exposure to physical inactivity varying between 70.4% for men and 75.7% for women in the year of 1990. This risk of exposure was similar in 2015. In men, the mortality rate from ischemic heart disease attributed to physical inactivity decreased in 2015 by approximately 24% around the world and 45% in Brazil. For women, this decrease was in 31% around the world and 45% in Brazil. The states of Southern and Southeastern Brazil presented lower mortality rates due to ischemic heart disease attributed to physical inactivity. If physical inactivity were eliminated in Brazil, mortality from ischemic heart disease would be reduced by 15.8% for men and 15.2% for women.

**CONCLUSIONS:**

Over 25 years, the risk of exposure to physical inactivity in Brazil did not change and was high compared to global estimates. The decrease in ischemic heart disease mortality results from the improvement of health services in Brazil and the control of other risk factors. Approximately 15% of deaths from ischemic heart disease in Brazil could be avoided if people met the recommendations for physical activity.

## INTRODUCTION

An epidemiological study^1^ evaluated the effects of physical inactivity on the burdens of chronic non-communicable diseases (NCD) worldwide, and they found approximately a 6.0% disease burden for coronary heart disease, 7.0% for type 2 diabetes, 10.0% for breast cancer, and 10.0% for colon cancer. This theme of attributing the burden of disease to physical activity was introduced into evidence in the early 2000s.

Even with this evidence, the level of physical activity in populations around the world continues to decline, prompting alarm from health services^2^. In Brazil, a 2014 survey of adult men and women from all capital cities showed that 64.7% of the adult population did not reach the recommended physical activity level^3^. This epidemic of physical inactivity results from several factors, such as the technological development that requires sedentary activities by the population^2^. Sedentary activities cause lower energy expenditure and lower chances of physical activity throughout the day^4^. Another factor that stands out is the growth of cities over the decades. In low- and middle-income countries, such as Brazil, the cities have grown without planning and spaces for promoting physical activity^5^.

Physical inactivity is a risk factor for several NCD (ischemic heart disease, arthritis, type 2 diabetes, some cancers, obesity, and stroke) which present a high burden of morbidity and mortality across the world^6^. Among NCD that have physical inactivity as one of the risk factors, ischemic heart disease stands out because it is responsible for one of the highest mortality rates attributable to cardiovascular diseases in Brazil^6^. In this sense, investigating how this disease with high mortality burden in Brazil is attributable to physical inactivity can help guide public policy.

The independent role of physical activity in the primary prevention of coronary heart diseases, such as ischemic heart disease, is well established and has been assessed in numerous reviews or meta-analyses^7^ ^,^ ^8^. Although all reviews agree that physical activity is associated with a 20.0% to 30.0% lower ischemic heart disease risk, no research has been conducted in Brazil to quantify the burden of mortality due to ischemic heart disease attributable to physical inactivity. This lack of information makes it difficult to monitor existing public policies to promote physical activity.

Brazil is a middle-income country that has recently improved its national health databases and implemented a series of surveys to better assess NCD risk factors, prevalence, and disease burden^9^. The 2015 Global Burden of Disease Study (GBD) has incorporated this more precise information into their internationally standardized approach for characterizing disease burden. Furthermore, to date, Brazil has engaged energetically in the WHO’s challenge to confront NCD^10^.

The aim of this study was to analyze if the trend of the burden of ischemic heart disease mortality attributed to physical inactivity in Brazil differs from the global estimates.

## METHODS

The 2015 GBD includes an annual assessment covering 195 countries and territories from 1990 to 2015. It covers 310 diseases and injuries, 2,619 sequelae, and 79 risk factors by age and sex. Detailed descriptions of the methodology and approach of the 2015 GBD have been published elsewhere^11–13^. In this study, the estimates presented were generated using the visualizations of the Institute for Health Metrics and Evaluation’s 2015 GBD^14^.

Cause of death was defined by international standards governing the reporting of death certificates, in which a single underlying cause is assigned by a physician. Ischemic heart disease was defined as an underlying cause of death across International Classification of Diseases (ICD) revisions, most recently in ICD-10 I20 to I25 and ICD-9 410 to 414^15^. Ischemic heart disease mortality was considered as the summation of four distinct disease sequelae: acute myocardial infarction, chronic stable angina, chronic ischemic heart disease, and heart failure due to ischemic heart disease^16^.

Vital registration, verbal autopsy and surveillance data were used to model ischemic heart disease. For Brazil, information from the Mortality Information System^14^ was used.

Physical activity was measured for adults older than or equal to 25 years of age, for durations of at least ten minutes at a time, across all domains of life (leisure or recreation, work, household, and transport). The frequency, duration, and intensity of activity were used to calculate the total metabolic equivalent (MET) in minutes per week. MET is the ratio of the working metabolic rate to the resting metabolic rate; one MET is equivalent to 1 kcal/kg/hour and is equal to the energy cost of sitting quietly. A MET is also defined as the oxygen uptake in ml/kg/min, with one MET equal to the oxygen cost of sitting quietly, which is approximately 3.5 ml/kg/min^13^.

We included surveys of the general adult population, performed using random sampling, that captured self-reported physical activity in all domains of life (leisure or recreation, work, household, and transport). For the global estimates, data were primarily derived from two standardized questionnaires, The Global Physical Activity Questionnaire and the International Physical Activity Questionnaire, although any other survey instrument that asked about the intensity, frequency, and duration of physical activities performed across all activity domains^13^ was included. For Brazil^17^, we consulted existing surveys such the Surveillance System for Risk and Protective Factors for Chronic Diseases by Telephone Survey (VIGITEL), Brazil World Health Survey, and the International Prevalence Study on Physical Activity. Due to the absence of a consistent relationship, on the individual level, between the amount of activity performed in each domain and total activity, it was not possible to use studies that included only recreational/leisure activities^13^. The physical activity level was categorized by total MET-minutes per week and the estimates were made for the subjects classified as inactive (< 600 METS-min/week)^18^.

The contribution of physical inactivity to the mortality by ischemic heart disease was estimated using a comparative risk assessment approach in which observed health outcomes are compared to those that would have been observed with a counterfactual set of exposure where no one is exposed^13^. For this, we used the Cause of Death Ensemble Modeling-CODEm (CODEm) that is used to estimate indicators by age, sex, country, year, and cause, and is an analytical tool that tests several possible statistical models of causes of death and creates a combined set of models that offers the best predictive performance. The software DisMod-MR 2.1, a meta-regression tool, is used for simultaneous estimates of incidence, prevalence, remission, disability, and also mortality attributable to risk factors, such as physical inactivity^13^. Modeling details can be found in the literature^11–13^.

The present study used the Summary Exposure Value (SEV – equation 1) concept. The SEV represents the prevalence of a disease weighted by the risk. The SEV scale varies from 0% to 100%, with 0% representing no risk of exposure and 100% representing maximum risk; a decline in SEV represents a reduced exposure, while an increase in SEV suggests the opposite. This measure was standardized by age. Information about physical inactivity in 1990 and 2015, by sex, was estimated. More details of SEV are available^13^. SEV was obtained by the equation 1:

$$\mathrm{Equation}\;1:\;SEV=\frac{\sum_{i=1}^{n}Pr_{i}RR_{i}–1}{RR_{max}–1}$$

Where:

Pr_i_ is the prevalence of the risk factor,

RR_i_ is the relative risk, and

RR_max_ is the observed maximum relative risk (between categories).

The study presents the SEV values for physical inactivity in 1990 and 2015, with 95% uncertainty intervals (95%UI), according to sex.

The population-attributable fraction (PAF) of death by ischemic heart disease due to physical inactivity was estimated^19^ using the following equation:

$$\mathrm{Equation}\;2:\;PAF=\frac{\sum_{i=1}^{n}P_{i}\left(RR_{i}–1\right)}{\sum_{i=1}^{n}P_{i}\left(RR_{i}–1\right)+1}$$

Where:

RR_i_ is the relative risk for exposure level i,

P_i_ is the proportion of the population in that exposure category, and

n is the number of exposure categories.

A systematic review of relevant epidemiological literature was conducted for the PAF estimates reported^20^. Due to considerable heterogeneity in the literature regarding physical activity metrics and the domain(s) covered, a methodologically intensive strategy was required to standardize the relative risk units to match those of the exposures^20^. More details about this analysis can be found in the literature^11^.

All calculations performed in GBD are repeated 1,000 times; these iterations are known as “draws”. Each draw takes a value from data distributions that reflect the sampling and non-sampling error of data inputs, as well as model uncertainty. All resulting estimates are presented with 95% uncertainty intervals (95%UI). The UI was defined by the 25th and 975th values of the 1,000 draws^11–13^.

The mortality rate standardized by age, the number of deaths due to ischemic heart disease attributable to physical inactivity, and the percent change information from 1990 to 2015 were estimated following the GBD study methodology. Based on this information, the standardized mortality rate due to ischemic heart disease attributable to physical inactivity in Brazil was compared with that from other high-, middle- and low-income countries. The countries of each category are defined by World Bank criteria and they have been grouped for this study^21^.

## RESULTS

**Figure 1:**
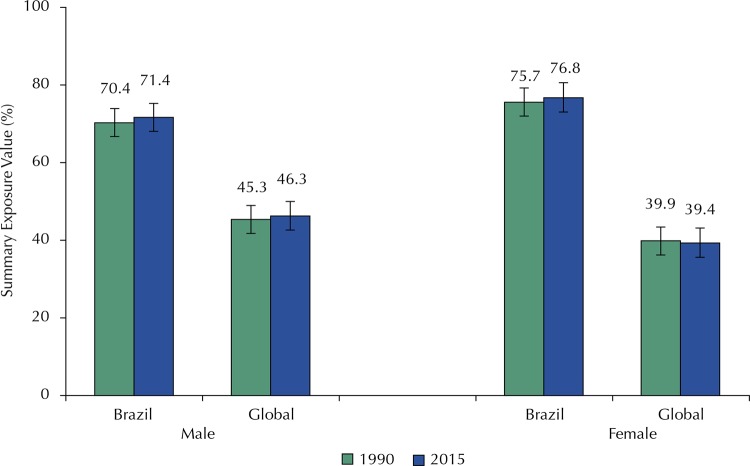
Synthesis of risk exposure (SEV) standardized by age for physical inactivity from 1990 to 2015 in Brazil and the world according to sex.

The Brazilian population was found to have a risk of exposure to physical inactivity varying between 70.4% for men and 75.7% for women in the year of 1990. This risk of exposure was similar in 2015. In 1990 and 2015, for both men and women, the risk of exposure to physical inactivity was higher in Brazil than around the world (Figure 1).

**Figure 2:**
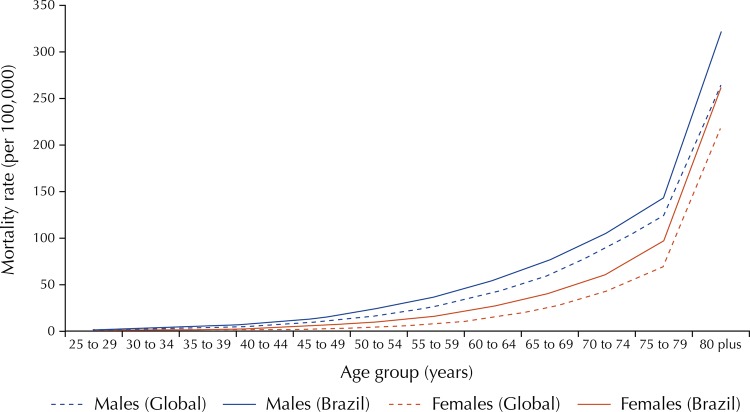
Global mortality rate and mortality rate in Brazil due to ischemic heart disease attributable to physical inactivity according to sex and age in 2015.

In men, the mortality rate due to ischemic heart disease attributable to physical inactivity decreased from 1990 to 2015, approximately, 24% and 45% around the world and in Brazil, respectively. In women, this decrease was 31% around the world and 44% in Brazil. Although this decreased mortality was more pronounced in Brazil compared to global rates, both in 1990 and 2015 these rates were higher in Brazil than around the world (Table 1; Figure 2).

**Table 1:** Age-standardized death rate due to ischemic heart disease attributable to physical inactivity in Brazil and worldwide (1990–2015).

Place	Age-standardized death rates (per 100,000)					Age-standardized death rates (per 100,000)				
	
	Male				% change (1990–2015)	Female				% change (1990–2015)
	
	1990		2015			1990		2015		
			
	Rate	95%UI	Rate	95%UI		Rate	95%UI	Rate	95%UI	
Global	23.31	15.65–31.43	17.88	12.22–24.01	-23.96	14.25	9.42–19.35	9.78	6.35–13.27	-31.06
Brazil	38.85	28.94–48.72	22.40	16.69–28.21	-42.99	25.24	18.93–31.70	13.91	10.36–17.42	-44.23
Acre	36.04	27.02–45.68	22.08	15.80–28.90	-39.28	22.35	16.55–28.84	13.47	9.39–18.26	-39.17
Alagoas	34.37	25.01–43.78	26.51	19.01–34.52	-23.89	26.02	18.65–32.97	17.29	12.16–23.06	-33.09
Amapá	27.76	20.57–35.62	20.22	13.50–28.68	-27.98	19.39	13.81–25.28	12.32	8.27–17.21	-36.47
Amazonas	30.84	22.98–39.57	19.13	13.52–25.75	-36.80	22.99	17.45–29.31	11.86	8.38–15.69	-48.27
Bahia	31.29	22.64–40.31	22.67	16.42–29.99	-26.51	23.03	16.74–29.40	15.37	11.08–20.62	-33.82
Ceará	29.75	21.23–38.33	26.49	18.70–35.81	-10.74	21.11	15.71–27.34	15.02	10.61–20.09	-28.20
Distrito Federal	29.84	21.62–38.41	15.26	11.03–20.19	-48.74	19.22	13.81–24.57	8.40	6.02–10.87	-56.78
Espírito Santo	36.24	26.15–46.92	21.20	15.19–28.10	-41.96	22.42	16.10–28.67	12.33	8.61–16.46	-44.45
Goiás	33.16	24.34–42.36	22.95	16.57–29.82	-30.02	24.58	18.48–30.88	15.28	11.04–19.73	-37.11
Maranhão	48.39	34.30–64.25	30.01	20.26–42.44	-37.97	20.99	15.10–27.67	18.42	12.46–26.12	-12.06
Mato Grosso	42.67	31.66–54.04	24.09	17.11–31.21	-43.52	31.12	22.77–39.50	15.53	10.83–20.36	-50.32
Mato Grosso do Sul	36.97	26.61–48.39	24.61	17.03–32.84	-33.15	25.57	18.27–32.72	13.81	9.68–18.09	-45.76
Minas Gerais	38.16	27.40–48.18	19.35	13.53–25.22	-49.16	24.98	18.21–32.21	12.17	8.75–15.83	-51.44
Paraná	39.23	28.44–50.59	21.90	15.92–28.95	-44.21	27.84	20.38–35.31	13.94	9.84–18.42	-49.38
Paraíba	34.31	25.47–43.55	32.60	23.25–42.71	-4.72	24.09	17.59–30.78	17.97	12.42–24.17	-25.79
Pará	32.76	23.96–42.18	23.24	15.94–31.56	-29.08	22.97	16.73–29.40	14.30	9.94–19.36	-37.39
Pernambuco	36.67	27.28–46.97	27.94	19.66–36.68	-23.08	24.71	17.87–31.34	16.82	11.69–22.46	-31.99
Piauí	31.85	23.16–40.92	27.42	19.75–36.89	-13.96	20.94	15.36–26.93	15.92	11.25–21.28	-23.71
Rio de Janeiro	50.98	37.83–65.19	25.18	17.44–33.56	-50.77	29.98	22.22–37.62	14.87	10.60–19.28	-50.85
Rio Grande do Norte	28.52	20.22–36.48	23.93	17.15–31.87	-16.21	19.46	14.56–24.82	13.89	9.75–18.29	-28.91
Rio Grande do Sul	42.83	30.78–55.47	20.54	14.24–27.30	-52.05	25.74	18.94–32.53	13.14	9.20–17.76	-48.74
Rondônia	44.23	32.99–56.28	25.75	18.73–33.47	-41.77	30.92	23.43–38.95	16.31	11.63–21.68	-47.86
Roraima	32.76	24.20–41.41	19.71	14.47–25.93	-39.37	20.03	14.81–25.58	11.02	7.95–14.40	-44.67
Santa Catarina	37.81	26.91–49.09	19.92	13.82–26.86	-47.13	25.49	18.58–32.50	12.71	8.91–16.79	-50.01
Sergipe	27.83	20.47–36.49	21.11	15.01–28.28	-24.51	19.81	14.30–25.61	13.82	9.61–18.55	-30.32
São Paulo	41.43	30.41–52.67	20.11	14.28–26.37	-51.71	26.24	19.11–33.35	12.74	9.03–16.63	-51.61
Tocantins	30.62	21.34–41.30	25.78	17.27–34.90	-15.18	22.66	15.49–31.16	16.28	11.18–22.31	-28.27

UI: uncertainty intervals

When analyzing the mortality rate due to ischemic heart disease attributed to physical inactivity in the Brazilian states, it can be observed that in men and women the greatest reductions (1990–2015) occurred in the states of the South and Southeast. In men, the lowest decrease in the mortality rate was observed in the state of Paraíba (-4.72%), where the mortality rate was higher in 2015. In women, this decrease was lower in the state of Maranhão (-12.06%), where the mortality rate was higher in 2015 (Table 1).

As shown in Table 2, if physical inactivity was eliminated from the world in 2015, the number of deaths due to ischemic heart disease would be decreased by 10.6% for men and 8.5% for women. In Brazil, this reduction would be more evident (15.8% for men and 15.2% for women). Physical inactivity was found to have an impact on death due to ischemic heart disease, which presented for all analyzed ages.

**Table 2:** Total number of deaths due to ischemic heart disease attributable to physical inactivity and population-attributable fraction in Brazil and worldwide according to age group (2015).

Age group	Mortality due to ischemic heart disease attributable to physical inactivity (2015)							

	Male (Global)		Female (Global)		Male (Brazil)		Female (Brazil)	
			
	Number of deaths	PAF: % (95%UI)	Number of deaths	PAF: % (95%UI)	Number of deaths	PAF: % (95%UI)	Number of deaths	PAF: % (95%UI)
All ages	515,021	10.6 (7.1–14.2)	343,666	8.5 (5.6–11.6)	17,018	15.8 (11.8–19.9)	13,413	15.2 (11.3–18.9)
25–29 years	3,804	15.9 (10.2–21.8)	1,063	11.9 (7.0–17.1)	92	23.3 (16.3–30.3)	44	27.7 (20.5–34.3)
30–34 years	6,496	14.0 (8.6–19.4)	1,680	10.9 (6.2–15.9)	219	23.4 (16.8–29.7)	96	26.2 (19.4–32.7)
35–39 years	9,455	12.9 (7.9–17.8)	2,310	10.1 (5.6–14.7)	371	22.9 (16.8–28.9)	160	24.6 (18.1–30.8)
40–44 years	15,516	12.6 (7.9–17.5)	3,735	9.8 (5.4–14.1)	603	22.2 (16.2–28.0)	271	22.9 (16.8–28.8)
45–49 years	24,394	12.2 (7.7–16.8)	6,420	9.6 (5.4–13.7)	948	21.0 (15.3–26.4)	458	21.4 (15.6–26.9)
50–54 years	34,134	11.4 (7.2–15.8)	9,725	9.3 (5.3–13.3)	1,464	19.5 (14.2–24.6)	694	19.9 (14.6–25.2)
55–59 years	45,020	11.3 (7.2–15.8)	14,060	9.0 (5.2–12.9)	1,787	18.2 (13.3–23.0)	874	18.8 (13.8–23.8)
60–64 years	61,001	11.4 (7.4–15.5)	23,224	8.9 (5.3–12.6)	2,043	17.4 (12.8–21.9)	1,165	17.9 (13.3–22.6)
65–69 years	59,332	11.4 (7.6–15.3)	27,909	8.9 (5.6–12.4)	2,089	16.3 (12.1–20.6)	1,358	16.9 (12.7–21.2)
70–74 years	65,143	10.4 (7.0–14.0)	36,884	8.1 (5.1–11.3)	1,937	15.1 (11.3–19.1)	1,443	15.6 (11.8–19.6)
75–79 years	61,385	9.8 (6.8–13.1)	43,383	8.0 (5.2–11.0)	1,841	13.8 (10.3–17.3)	1,724	14.2 (10.6–17.8)
≥ 80 years	129,334	9.3 (6.6–12.1)	173,267	8.5 (5.9–11.2)	3,619	12.5 (9.5–15.5)	5,120	12.9 (9.8–15.9)

PAF: Population-attributable fraction, i.e., the contribution of physical inactivity to death for ischemic heart disease; UI: uncertainty intervals

**Figure 3:**
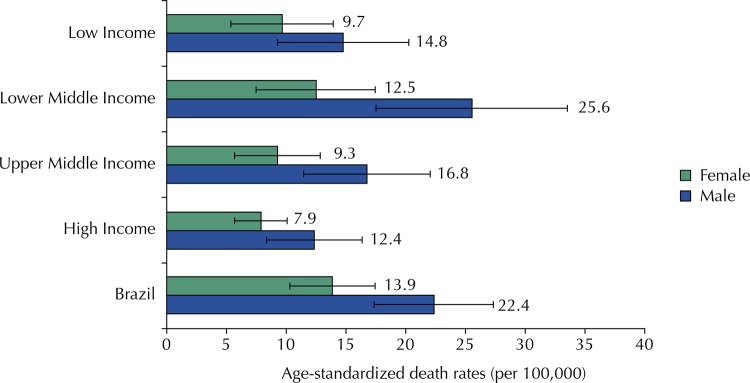
Age-standardized mortality rate due to ischemic heart disease attributable to physical inactivity in Brazil and groups of countries classified by income level as according to World Bank (2015).

Figure 3 shows that, in a global comparison, higher rates of mortality due to ischemic heart disease attributable to physical inactivity were reported in Brazil and middle- to low- income countries compared to high-income nations.

## DISCUSSION

The main finding of this study was that physical inactivity led to mortality due to ischemic heart disease in Brazilian adults and older people over the analyzed period (1990 to 2015). The results confirmed that the Brazilian population is more exposed to the risk of physical inactivity than the world population. Also, the mortality rate due to ischemic heart disease attributable to physical inactivity is correspondingly higher in Brazil than other countries around the world, regardless of differences in income level. Also, when analyzing the data of the Brazilian states, we observed that the greatest reductions from 1990 to 2015 in age-standardized death rates were in the states with better socioeconomic conditions.

The results of the present study agree with a meta-analysis of epidemiological studies that focused on physical activity as a primary preventative measure from coronary artery disease^7^. The meta-analysis, consisting of 33 studies, reported that individuals who engaged in the equivalent of 150 minutes/week of moderate-intensity leisure-time physical activity had a 14.0% (RR = 0.86, 95%CI 0.77–0.96) lower risk of coronary heart disease than those reporting no leisure-time physical activity. In addition, the meta-analysis found that those who 300 minutes/week of moderate-intensity leisure-time physical activity had a 20.0% (RR = 0.80, 95%CI 0.74–0.88) lower risk of coronary heart disease. The most surprising result of the meta-analysis was that individuals who were physically active at levels lower than the minimum amount recommended (< 150 minutes/week) had a risk of coronary heart disease significantly lower than those reporting no leisure-time physical activity. This result supports the assertion that some physical activity is better than none^7^.

Another meta-analysis using prospective cohort studies investigated both leisure-time physical activity and occupational physical activity^8^ and the associated risk of developing coronary heart disease through follow-up studies. The authors reported that men (RR = 0.76, 95%CI 0.70–0.82) and women (RR = 0.73, 95%CI 0.68–0.78) who met the physical activity guideline for health had a lower risk of developing cardiovascular disease than those with low levels of leisure-time physical activity. Similar results were found for occupational physical activity; people with a moderate level of occupational physical activity had lower risks of developing cardiovascular disease^8^. These findings support the results of the present study, which not only analyzed physical activity in the leisure period but in all domains (leisure, commuting, occupational, at home).

The risk of exposure of the Brazilian population to physical inactivity did not change from 1990 to 2015. However, the age-standardized death rates by ischemic heart disease attributable to physical inactivity decreased from 1990 to 2015. One explanation for this is that mortality by ischemic heart disease in Brazil has declined in those 25 years^6^ ^,^ ^22^. This decrease results from the improvement of health services in Brazil^22^ and the control of other risk factors for cardiovascular diseases, such as smoking^17^.

A disconcerting fact of the present study is that age-standardized death rates due to ischemic heart disease attributable to physical inactivity were higher in Brazil than worldwide for both sexes. This result suggests that more effort is required to promote physical activity in Brazil. The National Policy for Health Promotion was created in 2006 and represented a milestone in the policy of promoting physical activity in Brazil. Since the publication of this document, other strategies to promote physical activity have been launched, such as the National Plan of Physical Practices and Physical Activity and the Family Health Support Nucleus^23^ ^,^ ^24^. These initiatives provided greater awareness and access of the population to physical activity. However, few studies evaluating the impact, reach, and effectiveness of these policies can be found in the literature^24^ ^,^ ^25^. This limits the possibility for reformulation and for the implementation of new strategies to bring more effective results to the population in Brazil, a country with a continental dimension. Therefore, a frequent monitoring of these policies is necessary to reduce and combat the epidemic of physical inactivity.

One fact that may explain the high risk of exposure of the Brazilian population to physical inactivity and, consequently, the high number of deaths due to ischemic heart disease in adulthood is the high number of children and adolescents in Brazil who do not meet recommendations for physical activity. A survey monitoring physical activity in Brazilian youth found that almost 60.0% of individuals did not meet physical activity recommendations^26^. Therefore, the findings of this study may suggest that the promotion of physical activity should begin with younger age groups, especially for the prevention of NCD.

The Brazilian states with better socioeconomic conditions presented greater reductions (1990–2015) in mortality rates due to ischemic heart disease attributed to physical inactivity. This finding may reflect the lower prevalence of physical inactivity, obesity, and other risk factors in these regions compared to those with worse socioeconomic conditions, as evidenced by the VIGITEL^3^. An explanation for these discrepancies is the process of epidemiological transition in which Brazil is inserted. The more economically developed regions may already have better controlled NCD by means of risk factor control actions^3^.

This study provides important information about the impact of low levels of physical activity on mortality due to ischemic heart disease. The literature indicates that physical inactivity was responsible for 6.0% of coronary artery disease cases worldwide and 9.0% of premature mortality by any cause^1^. The findings of this study present the direct relationship between low levels of physical activity and mortality due to cardiovascular disease over time (1990–2015), demonstrating the case of Brazil, where physical inactivity is responsible for 15.0% of deaths due to ischemic heart disease.

This study analyzed all domains of physical activity (leisure, commuting, occupational, at home) to understand the benefits to people’s health and to inform the prevention of cardiovascular diseases. Furthermore, this study compared the mortality rate in Brazil with global estimates, considering national income levels, detailing the problem of low levels of physical activity across world populations. Finally, this study used the SEV measure to demonstrate the risk of exposure to physical inactivity in Brazil and around the world.

The first limitation of this study was the investigation of only one cardiovascular disease, which did not allow inferences regarding the impact of physical inactivity on diseases other than ischemic heart disease. Second, the effect of physical inactivity on other diseases such as cancer and diabetes has not been investigated. Third, the findings presented herein were based on surveys that estimate physical activity by questionnaires, which are considered subjective measures of physical activity and associated with measurement bias. Four, the non-stratification of physical activity by domains is another limitation. Finally, the estimates of physical activity made for the older population should be interpreted with caution, because the questionnaires used may not accurately measure physical activity in this population.

In this study, we decided to treat the terms ‘physical inactivity’ and ‘low levels of physical activity’ as synonyms to convey the message more clearly to the reader. The risk classification used was < 600 METS-min/week, as recommended by the literature^18^.

We can conclude that, over 25 years, the risk of exposure of the Brazilian population to physical inactivity has not changed and is high compared to the rest of the world. Physical inactivity is responsible for a significant number of deaths due to ischemic heart disease in Brazil and approximately 15.0% of these deaths could be avoided if people met the recommendations for physical activity. The mortality rate due to ischemic heart disease attributable to physical inactivity is higher in Brazil than in other countries, regardless of income level. The Brazilian states with better socioeconomic conditions presented greater reductions (1990–2015) in mortality rates due to ischemic heart disease attributed to physical inactivity.
